# Common misconceptions of ‘other effective area-based conservation measures’ (OECMs) and implications for global conservation targets

**DOI:** 10.1038/s44185-025-00079-5

**Published:** 2025-03-18

**Authors:** James A. Fitzsimons, Carolina Hazin, Joanna L. Smith

**Affiliations:** 1 The Nature Conservancy, Carlton, VIC Australia; 2 School of Life and Environmental Sciences, Deakin University, Burwood, VIC Australia; 3 School of Law, University of Tasmania, Hobart, TAS Australia; 4 The Nature Conservancy, London, UK; 5 The Nature Conservancy (Nature United), Fredericton, NB Canada

**Keywords:** Ecology, Ecology, Environmental sciences, Ocean sciences, Geography

## Abstract

The commitment to protect 30% of the Earth’s terrestrial, inland water, coastal and marine areas by 2030 under the Kunming-Montreal Global Biodiversity Framework has seen growing attention paid to ‘other effective area-based conservation measures’ (OECMs) to help achieve this target. However, there are a number of misconceptions of OECMs that commonly arise. We explore these misconceptions to aid in ensuring that OECMs are employed to meet their full potential.

## Introduction

In 2022, nations committed to achieving a global target of protecting at least 30% of the Earth’s terrestrial and inland water areas and coastal and marine areas by 2030, as part of the Convention on Biological Diversity’s Kunming-Montreal Global Biodiversity Framework (Target 3 – the ‘30 × 30 protection target’^1^). This ambitious commitment has seen rapidly growing attention to ‘other effective area-based conservation measures’ (OECMs) as an additional means to protected areas to achieve the target. The OECM term was introduced into the Convention’s lexicon in 2010, but only formally defined in 2018^2^, with IUCN guidance published the following year^3^. There has been increased encouragement from the academic and conservation communities to use OECMs to contribute to global conservation targets^4–6^.

Compared to the extensive literature on protected areas, consideration of OECMs has been sparse^7^; only a few papers have explored this at national^8–10^ and global levels^11^. In our experience at the interface between science and policy – where we actively work with national and subnational governments, non-government organisations, Indigenous groups and organisations, and other stakeholders on aspects of area-based conservation – there is excitement about the potential for OECMs to contribute to national and global biodiversity conservation targets. However, there are also common misconceptions of OECMs that frequently arise. Considering the importance of the 30 × 30 protection target for biodiversity conservation and the fast-approaching 2030 timeline, we outline some of these misconceptions below to aid in ensuring that OECMs are employed to meet their full potential.

## Common misconceptions of ‘other effective area-based conservation measures’

### Misconception 1. “If it is not a protected area on public land it must be an OECM”

The CBD and IUCN have recognized protected areas across a range of land and water governance and/or tenure types for many decades^12^. This includes Privately Protected Areas^13^, Indigenous and Community Conserved Areas (ICCAs) and Indigenous Protected Areas^14^ and, in some cases, shared arrangements. Some countries have applied and used the protected area categories that are suited to private, Indigenous or other lands and waters to achieve conservation targets^15,16^. However, other countries have not done this and have assumed that any area-based conservation measure that is different from the ‘conventional’ protected areas (e.g. public land protected through legal means) must therefore be an OECM. If an area is dedicated and managed for conservation, through legal or other effective means, and meets other criteria for a protected area, countries should classify it as such, even if requiring a new category, rather than an OECM (assuming consent is provided from the site's governance authorities).

### Misconception 2. “OECMs do not need to be long-term”

There is a misconception that OECMs can be applied to area-based conservation measures for a finite term (e.g. 25 years^17^) or with no guarantee of longevity (or proof of the likelihood of longevity)^7^. OECMs were envisioned to be long-term conservation measures for the landscape or seascape, not temporary components that would expire. The CBD^2^ defined OECMs as needing to be “governed and managed in ways that achieve positive and sustained long-term outcomes for the in situ conservation of biodiversity”. Likewise, by definition, protected areas should be established with the intention to manage and protect these areas in the long-term^12,17^.

### Misconception 3. “OECMs are quicker and easier to implement or recognize than protected areas”

A process to designate protected areas can take years and even decades. There is a misconception that it will be much quicker and easier to recognize (or create) and implement OECMs. In defining OECMs, the CBD^2^ stated they be “…governed and managed in ways that achieve positive and sustained long-term outcomes for the in situ conservation of biodiversity…”. Achieving positive and sustained long-term biodiversity outcomes is more stringent than the definition for a protected area, which emphasises the designation and management: “…recognized, dedicated and managed, through legal or other effective means, to achieve the long-term conservation of nature…”^12^. This is appropriate, as OECMs are still a relatively new concept, incorporating a range of different governance and management arrangements whose main purpose is often not primarily nature conservation. Unlike protected areas where areas reserved in broad categories (e.g. national parks, reserves, monuments) might automatically qualify, for OECMs each potential site needs to be assessed separately to determine whether a site meets the CBD definition and criteria, and the IUCN guidance for an OECM, which can be done with the aid of a toolkit^18^. There are additional requirements on OECMs^2^ such as “A monitoring system informs management on the effectiveness of measures with respect to biodiversity, including the health of ecosystems” and “Processes should be in place to evaluate the effectiveness of governance and management, including with respect to equity”. Like protected areas, OECMs recognized in lands or territories of Indigenous and traditional territories should require these peoples’ free, prior and informed consent, which is a process that can take some time. The administrative process to recognise an OECM can be also lengthy and follows, in some cases, more stringent criteria than for designating a protected area.

### Misconception 4. “OECMs need specific legislation, legislative recognition or a specific designation”

In most countries, protected areas are designated through specific legislation or related instruments, which is guided by a protected area policy. The ‘other’ in 'other effective area-based conservation measures' implies measures, mostly likely existing, that are ‘effective’ are sufficient, and these would not be the same measures required for protected areas. As such, ‘recognising’ these other measures that are effective is sufficient. Countries may choose to enable legislation or other mechanisms to formally recognize OECMs. Countries may also develop policies that will guide the achievement of the desired outcome in a coherent and effective manner. However, these are not requirements.

### Misconception 5. “Dedicated funding streams are required for OECMs”

The original intent of OECMs was a means to recognise ‘other’ area-based conservation measures that were *already* effective for biodiversity conservation, highly likely to continue this way in the long-term, and thus appealing to include towards global conservation targets. It was not necessarily envisaged that an ‘OECM network’ with new dedicated funding be established.

### Misconception 6. “If fisheries closures do not qualify as protected areas, they must be OECMs”

There has often been conjecture whether certain set-asides for fisheries management qualified as marine protected areas (MPAs). For a protected area designation the limitations in the purpose, intent and longevity are typically used to discount such closures as MPAs^19^. However, for OECMs, even though the primary purpose/intent does not need to be for conservation, biodiversity outcomes still must be achieved. There is increasingly significant interest in applying OECMs to existing fisheries closures or management areas to make potentially substantial contributions towards ocean 30 × 30 targets^20^. To meet the OECM criteria, fisheries closures would need to be long-term and whole ecosystem based, meaning that a closure for a single species in a management area allowing industrial fishing or seabed alteration would not be an OECM^21^. Long-term, fisheries areas closures that cover all species, and with a biodiversity outcome, may well qualify as OECMs^22^. Similar principles are true for agricultural and forestry activities.

## Implications for global conservation targets

Considering the scale of the biodiversity conservation challenges the world faces and the resultant ambition of the GBF, there is an understandable urgency for countries to expand protected area networks and identify OECMs to meet the 30 × 30 target. However, there is also a need to ensure that sites being considered for OECM recognition meet the criteria^23^ and are highly likely to deliver biodiversity outcomes over the long-term. Our experience is that there remains confusion within governments and other stakeholders about whether certain area-based conservation measures qualify as protected areas, OECMs, or neither. There is also uncertainty, despite past guidance^24^, in how ‘effectiveness’ for OECMs is defined, what the end goal of measuring effectiveness is (i.e. if it is ‘management effectiveness’ or ‘biodiversity outcomes’), and the tools needed to demonstrate ‘effectiveness’ in the long term. The recently released IUCN *Guidance on other effective area-based conservation measures (OECMs)*^25^ states, but is not explicit on this matter: “The choice of methods and frequency of monitoring will depend on the data required to understand the ecological values, the resources and capacity available, and the level of threat or rate of change at the site” and notes “Many monitoring and assessment tools exist … [which] … have different purposes, benefits and limitations, so an OECM governing authority will need to assess which one is appropriate”.

OECMs will likely be important for delivering biodiversity outcomes where protected area status cannot be achieved or may not be appropriate. However, we echo the caution of others^26^ in the potential misapplication of the OECM concept in the rush to meet global biodiversity targets. Addressing the misconceptions and building robust case studies should assist with increasing the use and application of OECMs to achieve biodiversity outcomes.

## Data Availability

No datasets were generated or analysed during the current study.
